# MRI Characteristics of Intraspinal Sparganosis: A Case Report

**DOI:** 10.2174/0115734056428861251217044412

**Published:** 2026-01-27

**Authors:** Yilan Zheng, Shuanghong Li, Xintian Wu, Tao Li

**Affiliations:** 1 Department of Radiology, Liuzhou Worker’s Hospital, Fourth Affiliated Hospital of Guangxi Medical University, Liuzhou, Guangxi, China; 2 Hospice Care Center, The South Campus of Liuzhou Workers' Hospital, Fourth Affiliated Hospital of Guangxi Medical University, Liuzhou, Guangxi, China

**Keywords:** Parasitic infection, Intraspinal sparganosis, Magnetic resonance imaging, Diagnostic approaches, Integrated treatment strategies, Preventive measures

## Abstract

**Background:**

Intraspinal sparganosis constitutes an uncommon parasitic infection. The absence of distinct clinical manifestations and imaging characteristics frequently leads to its misdiagnosis as a tumor, cyst, or hematoma.

**Case Presentation:**

In this study, we present a case involving a 57-year-old female patient with a history of consuming raw or undercooked frog meat and pork. Imaging studies identified an intraspinal occupying lesion. The patient subsequently underwent surgical intervention, which resulted in a pathological diagnosis of intraspinal sparganosis. Following this diagnosis, anthelmintic therapy was administered as part of the comprehensive treatment protocol.

**Conclusion:**

During the differential diagnosis of intraspinal space-occupying lesions, the intraspinal lesion observed on MRI plain scan appears as a solitary, irregular mass with abnormal signal characteristics. On T1-weighted imaging (T1WI), the lesion demonstrates isointense signal intensity; on T2-weighted imaging (T2WI), it displays mildly hyperintense signal, with markedly increased signal intensity on fat-suppressed T2WI. The lesion exhibits poorly defined margins and exerts a significant mass effect. Following contrast administration, the majority of lesions show marked, homogeneous, mass-like enhancement. Intraspinal sparganosis should be considered in the context of a comprehensive evaluation of the patient's MRI findings, medical history of potential exposure, and serological testing for parasitic antibodies. This integrated diagnostic strategy contributes to improved preoperative diagnostic accuracy, which in turn enhances treatment outcomes and prognosis.

## 1 INTRODUCTION

Sparganosis is a zoonotic parasitic infection caused by the larvae of various tapeworm species belonging to the family *Diphyllobothriidae*. While this disease occurs sporadically worldwide, it is more prevalent in South and Southeast Asian countries. Diphyllobothriid tapeworms undergo three primary developmental stages during their life cycle. Infected copepods are recognized as the first intermediate hosts, whereas frogs serve as the principal second intermediate hosts. When snakes, chickens, or ducks ingest infected frogs, they may function asparatenic hosts. The definitive hosts are typically carnivorous mammals, such as cats and dogs, in which the adult parasites reside within the intestinal tract [1, 2]. Humans most commonly act as paratenic hosts. The majority of sparganosis cases are associated with a history of applying raw frog meat to wounds or eyes, consuming undercooked frogs or snakes, or unintentionally ingesting water contaminated with infected copepods [3].

In human infections, spargana can localize in virtually any tissue except bone, with predilection sites including the ocular region, subcutaneous tissues of the limbs and trunk, oral and maxillofacial areas, and the central nervous system, particularly the brain. Visceral involvement is relatively uncommon, and spinal canal infestation is exceedingly rare, with only five documented cases reported in China between 2001 and 2025. Clinical manifestations depend on the anatomical location of the parasite and may include tissue destruction, visual impairment, epileptic seizures, paralysis, or even fatal outcomes; the pathological consequences of larval infection are substantially more severe than those caused by adult tapeworms [4, 5]. Spinal canal involvement may present with localized pain, spastic paralysis, increased muscle tone, pathological reflexes, perianal sensory deficits, disturbances in urinary and fecal continence, lower limb numbness, and progressive muscle weakness. Surgery remains the primary treatment for sparganosis, involving the removal of the scolex (head segment) of the sparganum and extraction of as many viable worms as possible. During surgical procedures, ethanol or lidocaine may be injected into the cyst or nodule to aid in parasite immobilization. Residual scolex fragments are recognized as the main cause of disease recurrence [6]. Although systemic pharmacological treatment primarily involves praziquantel, only one-seventh to one-fifth of the drug in the plasma can diffuse into the nervous system tissues surrounding larval lesions. Antiparasitic drugs, such as praziquantel, are generally considered ineffective in treating cerebral sparganosis caused by Spirometra larvae [7]. There are limited reports regarding intraspinal drug concentrations, and the therapeutic efficacy of pharmacological interventions remains inconclusive. Furthermore, case reports have indicated suboptimal efficacy of other antiparasitic drugs in treating parasitic infections of central nervous system.

## 2 CASE REPORT

A 57-year-old female presented with a 10-year history of low back pain and a 4-year history of left lower limb numbness and pain. She reported persistent dull pain in the lumbosacral region since 2013, without any identifiable trigger, which worsened with walking. Her mobility was mildly restricted, but there were no neurological deficits, fever, or systemic symptoms. In 2020, she developed left lower limb pain and numbness that improved with rest.

### 2.1 Magnetic Resonance Imaging Parameters at Our Institution

The examinations were performed using a Siemens Aera 1.5T MRI system equipped with a dedicated spinal coil. Standard protocols included sagittal T1-weighted imaging (T1WI), sagittal T2-weighted imaging (T2WI), sagittal fat-suppressed T2WI, axial T2WI, and contrast-enhanced fat-suppressed T1WI. All sequences were acquired with a slice thickness of 4 mm. The acquisition matrix sizes were 640×640 for sagittal T1WI, 768×768 for sagittal T2WI, and 512×512 for sagittal fat-suppressed T2WI. Contrast-enhanced imaging was performed following intravenous administration of gadolinium-DTPA (Gd-DTPA) at a dose of 0.1 mmol/kg body weight. An initial lumbar MRI in April 2020 revealed abnormal signals at the L4-S2 levels and the left S1/2 sacral foramen, prompting recommendations for enhanced imaging (Fig. **1**). The family declined surgical intervention and opted for conservative pain management. Over time, her symptoms recurred and progressively worsened. Upon readmission in June 2024, physical examination showed an ODI score of 60, a back pain VAS score of 6, and a leg pain VAS score of 4. There was tenderness over the L4-S1 region, mildly restricted spinal mobility, normal lower limb strength and sensation, and negative neurological tests. Her past medical history included consumption of undercooked frogs and pork. Repeat lumbar MRI in 2024 revealed intradural lesions at the L4-S2 levels compressing the cauda equina, with ill-defined margins (Fig. **2**). Laboratory tests showed elevated ESR (35 mm/h). During surgery, extensive adhesions between the lesion and nerve roots prevented complete resection. Histopathological analysis identified tapeworm parasites with eggs and chronic suppurative inflammation, with no evidence of malignancy (Fig. **3**). Serological testing confirmed Sparganum IgG positivity. Postoperatively, she received praziquantel (0.8 g TID × 5 days), dexamethasone, and mannitol. At the April 2025 follow-up, residual back and leg pain persisted but showed mild improvement. Final diagnosis was spinal canal sparganosis with chronic suppurative inflammation. Neurological examination findings remained stable relative to the preoperative status, and the patient was unable to undergo follow-up MRI due to familial financial constraints.

## 3 DISUSSION

Intraspinal sparganosis predominantly presents with localized pain and neuropathic leg pain secondary to nerve root compression. Lumbosacral pain is a frequent clinical manifestation, and the differential diagnosis includes intervertebral disc herniation, musculoskeletal disorders, intraspinal neoplasms, and other parasitic infections. Delayed diagnosis may result in postponed treatment initiation [8]. Definitive diagnosis of sparganosis typically relies on histopathological examination of surgically resected specimens. Macroscopically, the sparganum appears as ribbon-like, milky-white structures resembling adult cestodes, characterized by transverse folds and a non-segmented body. When morphological integrity is compromised or visual identification is inconclusive, microscopic histopathological analysis becomes essential. Chi *et al*. [9] examined multiple tissue sections and demonstrated that host tissue reactions to sparganum are closely associated with parasite migration and are typically localized in proximity to the organism. Histological findings commonly include eosinophilic abscesses or granulomas containing extensive necrotic debris, occasionally accompanied by sinus tract remnants [10]. Preoperative imaging and serological testing can provide valuable information regarding lesion localization and etiology. Serological assays exhibit high sensitivity, strong specificity, rapid turnaround time, and cost-effectiveness in diagnosing sparganosis [11], although cross-reactivity with other helminthic infections may occur [12]. Immunodetection of *Spirometra mansoni* antigens primarily involves four categories: crude soluble antigens, excretory-secretory antigens, purified native antigens, and recombinant antigens. Furthermore, routine hematological evaluation can aid in the diagnosis of *S. mansoni* infection, with peripheral blood eosinophilia serving as a supportive indicator of helminthic infestation [13, 14]. Advances in molecular diagnostics have introduced DNA sequencing as a novel tool for confirming sparganosis [15, 16]. Recently, Hu *et al*. [17] successfully applied metagenomic next-generation sequencing (mNGS) to identify parasite species directly from pathological specimens. Imaging modalities play a critical role in auxiliary diagnosis and include computed tomography (CT), magnetic resonance imaging (MRI), and ultrasonography, selected according to the anatomical location of the parasite. Ultrasonography is particularly effective for cutaneous and superficial organ involvement, whereas CT and MRI are preferred for deep-seated lesions and central nervous system infections. As a non-invasive modality, MRI allows for serial evaluations; dynamic changes or migratory patterns of lesions may indicate the presence of viable parasites, thereby improving diagnostic accuracy. Moon *et al*. [18] first proposed MRI-based criteria for cerebral sparganosis; however, imaging characteristics of intraspinal sparganosis remain poorly documented. Intraspinal lesions are often initially suspected to be benign schwannomas, which typically appear on MRI as well-circumscribed, encapsulated masses with round or oval morphology. Signal intensity usually demonstrates iso- to hypointense signal on T1-weighted imaging (T1WI) and mildly hyperintense to hyperintense signal on T2-weighted imaging (T2WI), sometimes with cystic degeneration. Contrast-enhanced scans generally show marked homogeneous or heterogeneous enhancement of solid components [19], with clear demarcation from the spinal cord and cauda equina. In this case of intraspinal sparganosis, MRI in April 2020 revealed a patchy lesion within the L4–S2 spinal canal extending into the left S1/2 sacral foramen, exhibiting isointense signal on T1WI, mildly hyperintense signal on T2WI, and hyperintense signal on fat-suppressed imaging, measuring approximately 1.7 cm × 1.4 cm × 6.0 cm, with heterogeneous signal intensity. Follow-up MRI in June 2024 demonstrated a mass-like lesion in the L4–S2 spinal canal, measuring approximately 1.8 cm × 1.5 cm × 6.2 cm, with isointense signal on T1WI, mildly hyperintense signal on T2WI, and hyperintense signal on fat-suppressed imaging. The lesion exhibited ill-defined margins and significant contrast enhancement, consistent with inflammatory changes induced by sparganum. Compared to benign intraspinal tumors, the most distinctive imaging features of sparganosis include poorly delineated borders and homogeneous enhancement on contrast-enhanced imaging. Although certain malignant intraspinal tumors may share similar radiological appearances, they are typically characterized by rapid progression and distant metastasis, which can be differentiated based on clinical history and tumor marker profiles. Current management of intraspinal sparganosis primarily consists of surgical excision; however, pharmacological therapy lacks robust evidence of efficacy. Complete resection is frequently hindered by dense adhesions to neural structures, leading to suboptimal outcomes and incomplete symptom resolution. Therefore, preventive strategies should emphasize food safety education and the elimination of high-risk behaviors, such as consuming raw amphibians or reptiles and applying raw meat topically. This report has several limitations, including its nature as a single-case study and the patient’s inability to undergo further follow-up or additional treatment due to familial financial constraints.

## CONCLUSION

Due to the low incidence of intraspinal sparganosis, there are currently no standardized treatment guidelines. A review of the literature indicates that lesions predominantly occur in the sacrococcygeal region, where complete surgical resection is often difficult to achieve, and pharmacological interventions lack evidence-based validation, leaving treatment largely dependent on empirical approaches. Pathological, immunological, molecular biological, and imaging examinations each have distinct advantages and limitations. An integrated application of these diagnostic modalities can effectively compensate for the shortcomings of individual methods [20]. Combined with a history of epidemiology plan scan image from 2024. In patients with immunological testing for parasitic antibodies, intraspinal sparganosis should be considered in the differential diagnosis of any intraspinal space-occupying lesion identified on imaging. Definitive diagnosis *via* pathological confirmation of sparganosis is essential to prevent misdiagnosis and inappropriate treatment, thereby improving therapeutic outcomes and prognosis while minimizing unnecessary consumption of medical resources.

## Figures and Tables

**Fig. (1):**
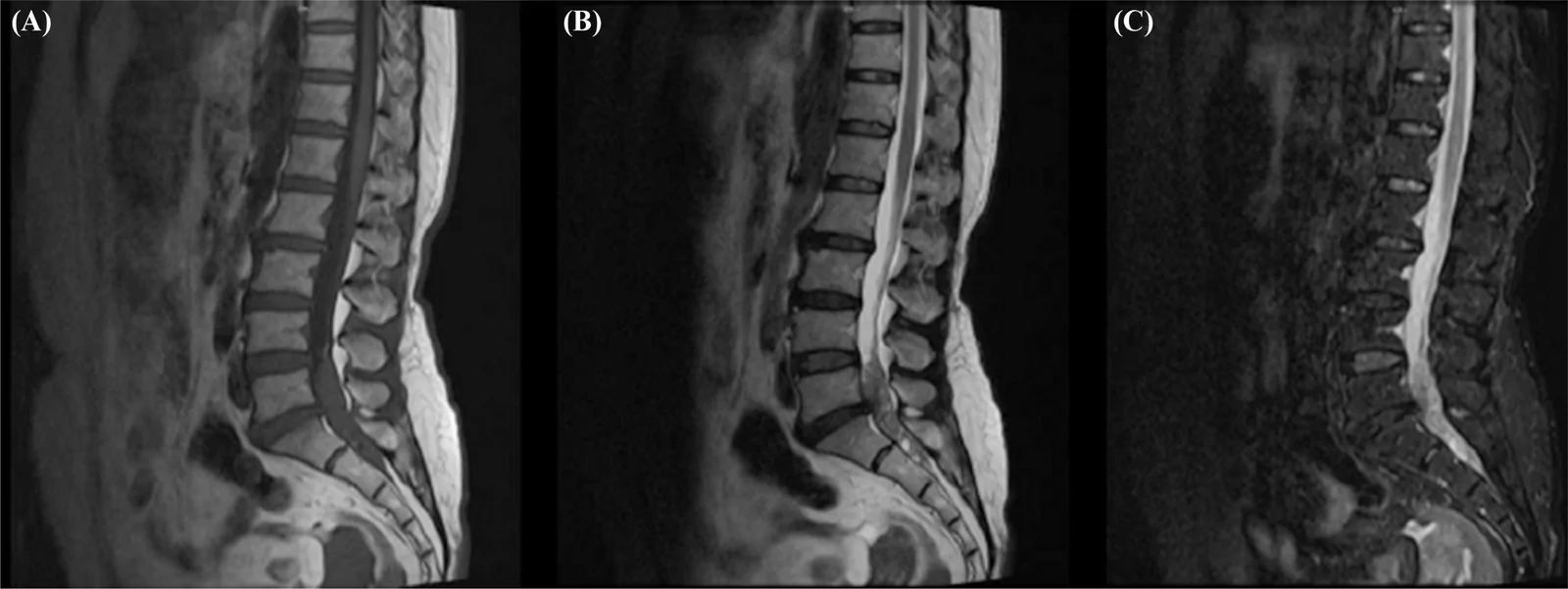
Magnetic resonance imaging characteristics of intraspinal sparganosis, April 2020. (**A**), Sagittal T1-weighted imaging reveals a patchy isointense signal within the lesion. (**B**), Sagittal T2-weighted imaging shows a slightly hyperintense, patchy signal. (**C**), Sagittal T2 fat-suppressed imaging demonstrates a hyperintense lesion with heterogeneous signal intensity and ill-defined margins. The mass compresses the cauda equina and left sacral nerve roots, with no evidence of osseous destruction.

**Fig.(2):**
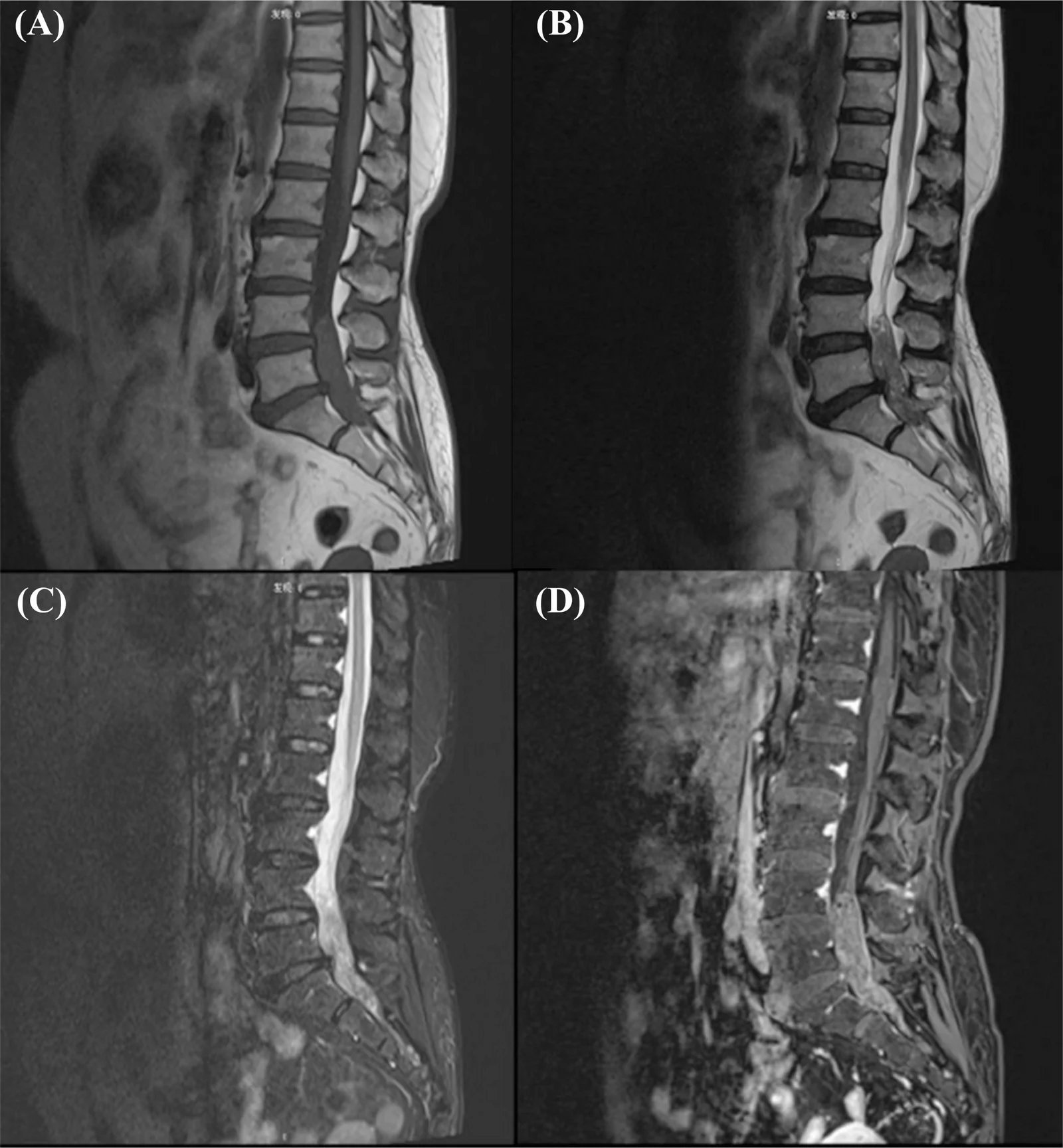
Magnetic resonance imaging characteristics of intraspinal sparganosis in June 2024. (**A**). On the sagittal T1-weighted image, the mass exhibits a patchy isointense signal. (**B**). On the sagittal T2-weighted image, the mass demonstrates a slightly hyperintense patchy signal. (**C**), On the sagittal T2 fat-suppressed weighted image, a hyperintense lesion is observed. (**D**), On the sagittal T1 fat-suppressed contrast-enhanced image, the tumor exhibits significant enhancement. The tumor presents with heterogeneous signal intensity and indistinct margins, showing slight enlargement compared to April 2020. Compression of the cauda equina and left sacral nerves is evident, with no evidence of bone destruction.

**Fig. (3):**
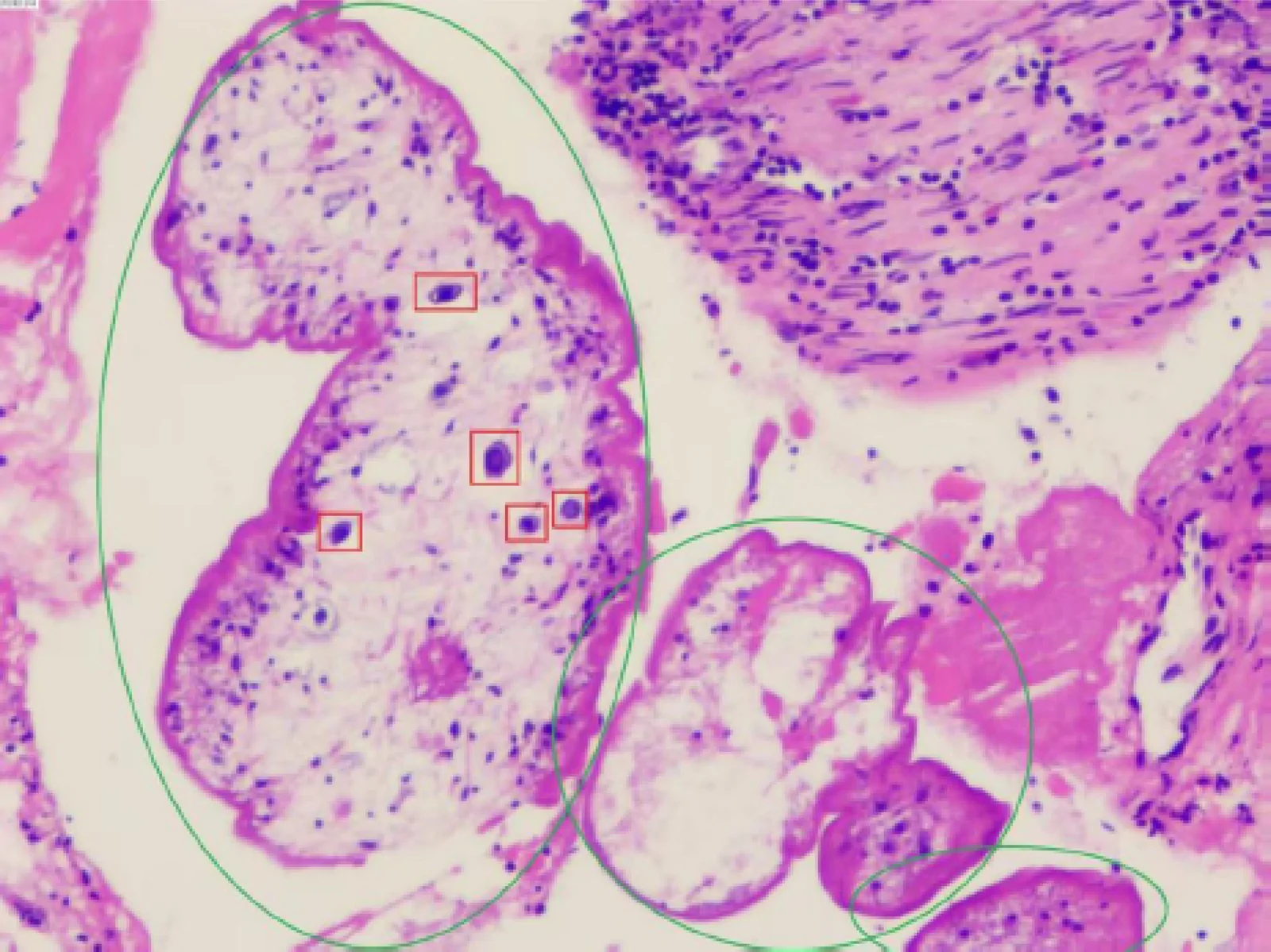
Pathology. The cross-section of the worm body is circled in green, and the cross-section of the egg is marked in red.

## Data Availability

All data generated or analyzed during this study are included in this published article.

## LIST OF ABBREVIATIONS

MRI: Magnetic resonance imaging
VAS: Visual analogous scale
DWI: Diffusion-weighted Imaging
Ct: Computed tomography
mNGS: Metagenomic next-generation sequencing

## STANDARDS OF REPORTING

CARE guidelines were followed.
